# Association of the sEH gene promoter polymorphisms and haplotypes with preeclampsia

**DOI:** 10.5937/jomb0-27745

**Published:** 2020-10-02

**Authors:** İsmail Sarı, Hatice Ökten, Çağdaş Aktan, Esra Cihan

**Affiliations:** 1 Niğde Omer Halisdemir University, Faculty of Medicine, Department of Medical Biochemistry, Niğde, Turkey; 2 Beykent University, Faculty of Medicine, Department of Medical Biochemistry, Istanbul, Turkey; 3 Beykent University, Faculty of Medicine, Department of Medical Biology, Istanbul, Turkey; 4 Niğde Omer Halisdemir University, Faculty of Medicine, Department of Obstetrics and Gynaecology, Niğde, Turkey

**Keywords:** epoxyeicosatrienoic acids, preeclampsia, soluble epoxide hydrolase, polymorphism, gene promoter, promoter gena, polimorfizam, rastvoriva epoksidna hidrolaza, preeklampsija, epoksi-eikosatrienske kiseline

## Abstract

**Background:**

The epoxyeicosatrienoic acids (EETs) have antihypertensive, anti-inflammatory, and organ protective properties and their circulation levels are related to hypertension, diabetes mellitus, cardiovascular diseases, and preeclampsia. Soluble epoxide hydrolase (sEH) catalyses the degradation of EETs to less biologically active dihydroxyeicosatrienoic acids. Here, we sequenced the promoter region of *EPHX2* to investigate the association between promoter sequence alterations that we thought to affect the expression levels of the enzyme and preeclampsia (PE).

**Methods:**

Nucleotide sequencing of the promoter region of the *EPHX2*, spanning from position -671 to +30, was performed on 100 pregnant women with PE and, 20 or more weeks pregnant normotensive, healthy women (n=100).

**Results:**

Pregnant women who carry rs4149235, rs4149232, rs73227309, and rs62504268 polymorphisms have 4.4, 2.4, 2.3, and 2.8 times significantly increased risk of PE, respectively. CCGG (OR: 3.11; 95% CI: 1.12-8.62) and CCCA (OR: 0.45; 95% CI: 0.36-0.55) haplotypes were associated with an increased and decreased risk of PE, respectively.

**Conclusions:**

Four SNPs (rs4149232, rs4149235, rs73227309, and rs62504268) in the promoter region of the *EPHX2*, and CCGG and CCCA haplotypes of these 4 SNPs were significantly associated with PE. These SNPs in the promoter region may affect sEH expression and thus enzyme activity and may play a role in PE pathogenesis by causing individual differences in EET levels. However, future studies are needed to confirm our findings and examine the effect of these SNPs on the sEH expression and/or enzyme activity.

## Abstract (sr)

**Uvod:**

Epoksi-ekosatrienoične kiseline (EET) imaju antihipertenzivna, protivupalna i zaštitna svojstva organa i nivoi njihove cirkulacije povezani su sa hipertenzijom, dijabetesom, kardiovaskularnim bolestima i preeklampsijom. Rastvoriva epoksidna hidrolaza (sEH) katalizuje razgradnju EET-a na biološki manje aktivne dihidroksiekosatrienoične kiseline. Uradili smo analizu sekvenci regiona promotera EPHKS2 da bismo istražili povezanost izmena sekvenci promotera za koje smo mislili da utiču na nivoe ekspresije enzima i preeklampsije (PE).

**Metode:**

Nukleotidno sekvenciranje promoterske regije EPHKS2, koje se proteže od položaja -671 do +30 izvršeno je na 100 trudnica sa PE, kao i na ženama koje su bile trudne 20 ili više nedelja, a koje su bile zdrave i sa normalnim krvnim pritiskom (n = 100).

**Rezultati:**

Trudnice koje nose rs4149235, rs4149232, rs73227309 i rs62504268 polimorfizme imaju 4,4, 2,4, 2,3 i 2,8 puta značajno povećan rizik od PE. CCGG (OR: 3,11; 95% CI: 1,12-8,62) i CCCA (OR: 0,45; 95% CI: 0,36-0,55) haplotipovi su povezani sa povećanim i smanjenim rizikom od PE.

**Zaključak:**

četiri SNP-a (rs4149232, rs4149235, rs73227309 i rs62504268) u promoterskoj regiji *EPHKS2*, i CCGG i CCCA haplotipovi ova 4 SNP-a su u značajnoj vezi sa PE. Ovi SNP-ovi u promoterskoj regiji mogu uticati na ekspresiju sEH, a time i na aktivnosti enzima i mogu igrati ulogu u patogenezi PE uzrokujući pojedinačne razlike u nivoima EET-a. Međutim, potrebno je sprovesti dodatne studije u budućnosti da se potvrde naša otkrića i da se ispita uticaj ovih SNP-a na sEH ekspresiju i/ili aktivnost enzima.

## Introduction

Preeclampsia (PE) is a systemic and complex syndrome characterised by a new onset of hypertension and proteinuria or new onset of hypertension and significant end-organ dysfunction with or without proteinuria after the 20^th^ week of pregnancy or sometimes during the postpartum period. PE is a leading cause of maternal and fetal mortality and morbidity and, occurs in about 7.5% of pregnant women [1]. Although the aetiology of PE is not entirely understood, placental, maternal, immune, and genetic factors have an essential role in the pathogenesis of this disease [2]. There is a consensus among researchers that endothelial dysfunction plays a vital role in the pathogenesis of PE [3] [4]. Endothelial dysfunction refers to impairment of the endothelium-dependent relaxation caused by an imbalance between the vasodilators and vasoconstrictors that are necessary mediators in the local control of blood flow [2]. Arachidonic acid and its metabolites play an important role in the regulation of vascular tonus. Arachidonic acid is oxidised by the CYP monooxygenase to epoxyeicosatrienoic acids (EETs), vasodilator, natriuretic and anti-inflammatory substances and they act as an endothelial-derived hyperpolarisation factor in the various vascular beds [5] [6]. These molecules are metabolised to the correspondent dihydroxyeicosatrienoic acids (DHETs), mostly inactive compounds, by soluble epoxide hydrolase (sEH; EC 3.3.3.2) [6] [7] [8]. Previous studies suggested that altered EET levels contribute to the pathophysiology of hypertension [9], coronary heart disease [10], ischemic stroke, and vascular dysfunction [11]. CYP monooxygenases and sEH activities and/or expression play a critical role in the control of EET levels. Therefore, genetic polymorphisms that affect the activity or the expression level of these enzymes may be associated with the diseases mentioned above [9] [10] [11] [12].

It was suggested that inhibition of sEH prevents EET degradation, and enhances their biological activities. Thus, interest has been raised for the use of drugs targeting the sEH for the treatment of myocardial infarction [12], atherosclerosis [13], ischemia [14], inflammation-related pathologies [15] and metabolic syndrome [16], and hypertension [17]. Furthermore, it was found that EETs regulate the uterine blood flow in pregnancy, and their levels are reduced in PE and pregnancy-induced hypertension [18] [19] [20]. Moreover, in our previous work, we determined that hypomethylation of the *EPHX2* promoter that may cause higher expression of sEH, and K55R polymorphism that gives rise to an increase of the enzyme activity, are associated with significantly increased risk of PE [21]. Thus, we consider that individual differences in the promoter region of the gene encoding the sEH enzyme that affects the expression level may play a role in the pathogenesis of PE as well as the promoter hypomethylation and also functional polymorphisms that affect enzyme activity. Although it is clearly known that some of the single nucleotide polymorphisms (SNP) also can cause alterations in gene expression, there is a lack of information about the frequency of the SNPs in the promoter region of the *EPHX2* in PE patients and their association with PE. Here we sequenced the *EPHX2* promoter region of the PE patients to investigate the relationship between PE and promoter sequence differences of the gene encoding the sEH.

## Materials and Methods

### Study population

One hundred pregnant women with PE and 100 healthy, normotensive, 20 or more weeks pregnant women without chronic hypertension and diabetes were included in the study. PE was diagnosed as the presence of new-onset hypertension (systolic blood pressure ≥ 140 mmHg or diastolic blood pressure ≥ 90 mmHg) plus either proteinuria (proteinuria ≥ 0.3 grams in a 24-hour urine specimen or protein: creatinine ratio ≥ 0.3) or end-organ dysfunction (platelet count < 100,000/microliter, serum creatinine > 0.09 mmol/L or doubling of the serum creatinine, elevated serum transaminases to twice normal concentration) after 20 weeks of pregnancy in a previously normotensive woman [22]. The Ethical Committee approved the study of Cumhuriyet University (2015-05/08). Informed consent was obtained from all subjects.

### DNA Isolation

Genomic DNA was extracted from peripheral blood lymphocytes using the Qiagen DNA isolation kit (QIAamp® DNA Mini 250). DNA concentration and purity of isolated DNA samples were measured by MaestroNano Spectrophotometer (Maestrogen, USA).

### Sanger sequencing

For the sequencing of the promoter region of *EPHX2*, we utilised a reference sequence from the eukaryotic promoter database (nt -671 to +30) (https://epd.vital-it.ch/index.php). For mutation detection, the promoter region of the *EPHX2* was amplified by polymerase chain reaction (PCR). The primer pairs for PCR were F:5' GAGATTGAAATC-GAAGTATTCTGGG-3', R: R:5' AGCTAACCTGGGA-GATGCG-3'. The PCR products were visualised on a 1% agarose gel and were then extracted from the gel by using the DNA extraction kit GelSV (GeneAll Cat no: 102-150). The purified PCR products were used as templates, and the PCR primers used for amplification were also used as sequencing primers. BigDye terminator (v3.1) cycle sequencing kit and 3130 Genetic Analyser (Applied Biosystems, USA) was used for sequencing and analysis, respectively. FinchTV 1.4.0 (GeospizaInc Seattle, WA) was used for the interpretation of sequencing chromatograms. Data were also analysed with Chromas Lite 2.0 (Technelysium Pty Ltd., Australia) and seqscape v2.6 program (Applied Biosystems), and were compared with the reference sequences. Samples with poor sequencing quality were excluded from the study, and further statistical analyses.

### Statistical analysis

Statistical analyses were performed using the SPSS software (Statistical Package for the Social Sciences, version 15.0, SPSS, Inc., Chicago, IL, USA). Means of age, gravidity, parity, diastolic, and systolic blood pressures were analysed by independent-samples t-test. Genotypes were analysed in study groups by 2 test. As an estimation of the relative risk of the disease, the odds ratio (OR) was calculated based on 95% confidence intervals (CI). *P*-values < 0.05 were considered to be statistically significant. Pairwise linkage disequilibrium (LD), haplotype frequencies and haplotype associations between four SNPs were carried out using Haploview 4.2 software [23].

## Results

The demographic features of the study group were shown in Table 1. No statistically signi cant difference was determined in terms of mean age, gravida, and parity between patients and controls (p > 0.05), while the difference between the means of systolic and diastolic blood pressure was statistically signi cant (p < 0.05).

**Table 1:** Demographics and some clinical features of thestudy group. *p < 0.05 confirmed as significant; OR, odds ratio; CI, confidence interval

	Patient (N = 79)	Control (N = 79)	p value
Age (x̄ ± S)	29.6 ± 7.0	28.0 ± 6.6	0.167
Gravida (x̄ ± S)	1.8 ± 1.1	1.8 ± 0.8	0.56
Parity (x̄ ± S)	1.7 ± 0.8	1.6 ± 0.9	0.52
Systolic blood pressure (mmHg; x̄ ± S)	160.1 ± 9.2	110.2 ± 20.1	0.0001*
Diastolic blood pressure (mmHg; x̄ ± S)	104.4 ± 11.2	66.4 ± 6.8	0.0001*

The polymorphic sites within the *EPHX2* promoter that we found an association with PE, were given in Table 2 and Table 3. All of the detected sequence differences in the *EPHX2* promoter were SNPs (Ensemble). As can be seen in Table 2, genotype distribution and allele frequencies of rs62504268, rs72473923, and rs4149235 polymorphisms were significantly different among patients and controls. Although the genotype distribution of rs73227309 was not significantly different in patients and controls, its allele frequency was significantly different between the two groups.

**Table 2:** Allele and genotype frequencies of SNPs in the *EPHX2* promoter region.

Polymorphism	Patient (N = 79), n (%)	Control (N = 79), n(%)	p-value
rs62504268 (NM_001256482.1:c.460G>A)			
GG	56 (70.9)	69 (87.4)	
GA	20 (25.3)	9 (11.4)	
AA	3 (3.8)	1 (1.2)	0.035*
Allele frequency			
G	132 (83.5)	146 (92.4)	
A	26 (16.5)	12 (7.6)	0.015*
rs4149232 (NM_001256482.1:c.-687T>C)			
TT	42 (53.2)	58 (73.4)	
TC	36 (45.6)	21 (26.6)	
CC	1 (1.2)	0 (0)	0.023*
Allele frequency			
T	120 (75.9)	137 (86.7)	
C	38 (24.1)	21 (13.3)	0.014*
rs4149235 (NM_001256482.1:c.681G>A,C)			
GG	39 (49.4)	64 (81,0)	
GC/GA	39 (49.4)	14 (17.8)	0.0001*
CC/AA	1 (1.2)	1 (1.2)	
Allele frequency			
G	117 (74.1)	142 (89.9)	
C/A	41 (25.9)	16 (10.1)	0.0001*
rs73227309 (NM_001256482.1:c.-575G>C)			
GG	56 (70.8)	67 (84.8)	
GC	19 (24.1)	10 (12.7)	
CC	4 (5.1)	2 (2.5)	0.105
Allele frequency			
G	131 (82.9)	144 (91.1)	
C	27 (17.1)	14 (8.9)	0.030*

**Table 3:** Risk estimates for SNPs in the *EPHX2* promoter region *p < 0.05 confirmed as significant; OR, odds ratio; CI, confidence interval.

Polymorphism	Patient (N = 79)%	Control (N = 79)%	OR (95% CI)
rs62504268			
GG	70.9	87.4	Reference
GA +AA	29.1	12.6	2.83 (1.24–6.44)*
Allele frequency			
G	83.5	92.4	Reference
A	16.5	7.6	2.40 (1.16–4.94)*
rs4149232			
TT	53.2	73.4	Reference
TC +CC	46.8	26.6	2.43 (1.25–4.74)*
Allele frequency			
T	75.9	86.7	Reference
C	24.1	13.3	2.07 (1.15–3.74)
rs4149235			
GG	49.4	81	Reference
GC/GA + CC/AA	50.6	19	4.38 (2.14–8.94)*
Allele frequency			
G	74.1	89.9	Reference
C/A	25.9	10.1	3.11 (1.66–5.82)*
rs73227309			
GG	70.8	84.8	Reference
GC + CC	29.2	15.2	2.29 (1.05–5.02)*
Allele frequency			
G	82.9	91.1	Reference
C	17.1	8.9	2.12 (1.07–4.22)*

Logistic regression analyses showed that rs62504268, rs4149232, rs4149235 and, rs73227309 polymorphisms in the promoter region of *EPHX2* were significantly associated with PE. Moreover, mutant alleles of these four SNPs were significantly associated with increased PE risk (p < 0.05, Table 3). All genotype and allele frequencies were in Hardy-Weinberg equilibrium in the control group.

**Figure 1:**
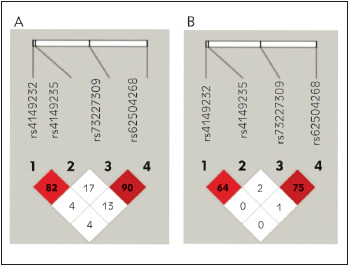
Linkage disequilibrium tests for four SNPs atthe *EPHX2* promoter. Linkage disequilibrium coefficients(|D’| (A) and *r* ^2^ (B)) of the four SNPs.

Furthermore, we analysed linkage disequilibrium (LD) of the SNPs in the *EPHX2* promoter. According to the linkage disequilibrium analysis, D' values between rs4149232 and rs4149235, rs4149232 and rs73227309, rs73227309 and rs62504268, rs4149235 and rs73227309, rs4149235 and rs62504268, rs73227309 and rs62504268 were 82, 4, 4, 17, 13, and 90%, respectively (Figure 1A). In addition, the linkage scores, according to *r* ^2^ analysis between the mentioned polymorphisms were 64, 0, 0, 2, 1, 75%, respectively Figure 1B).

According to D' and r^2^ values the strongest LD was observed between two SNPs located in the promoter of the *EPHX2*: rs73227309-rs62504268 (D' = 0.908; r^2^ = 0.756) and rs4149232-rs4149235 (D' = 0.82; r^2^ = 0.645) (Table 4, and Figure 1).

**Table 4:** The results of linkage disequilibrium in the *EPHX2* promoter. L1 and L2 are the two loci; D’ is the value of D prime between the two loci; LOD is the log of the likelihood odds ratio, a measure of confidence in the value of D’; r2 is the correlation coefficient between the two loci; CIlow is 95% confidence lower bound on D’; CIhi is the 95% confidence upper bound on D’.

L1	L2	D’	LOD	r^2^	CIlow	CIhi
rs4149232	rs4149235	0.82	23.63	0.645	0.72	0.89
rs4149232	rs73227309	0.042	0.05	0.001	-0.01	0.23
rs4149232	rs62504268	0.046	0.05	0.001	-0.01	0.24
rs4149235	rs73227309	0.171	0.78	0.02	0.03	0.34
rs4149235	rs62504268	0.136	0.46	0.011	0.01	0.31
rs73227309	rs62504268	0.908	28.32	0.756	0.8	0.97

Lastly, we performed haplotype analysis for the 4 SNPs in the *EPHX2 *promoter. Identified haplotypes and their frequencies in patients and controls are shown in Table 5. According to the haplotype analysis, haplotype frequencies of TGGG, CCGG, CGGG, and CCCA were significantly different between patients and controls. Univariate analysis showed an association between significantly increased risk of PE and CCGG haplotype, whereas the decreased risk of CCCA haplotype (p < 0.05). However, there was no statistically significant association between PE and the other 2 haplotypes (p > 0.05).

**Table 5:** Association between *EPHX2* promoter haplotypes and PE. OR, odds ratio; CI, confidence interval; SNPs, single nucleotide polymorphisms

Haplotypes	SNPs				Frequency		Chi^2^	p	OR (%95 CI)
	rs4149232	rs4149235	rs73227309	rs62504268	Patient(N = 79)	Control(N = 79)			
1	T	G	G	G	0.62	0.77	8.45	0.004*	Reference
2	C	C	G	G	0.19	0.07	9.79	0.002*	3.11* (1.12–8.62)
3	T	G	C	A	0.10	0.06	2.14	0.143	1.99 (0.61–6.47)
4	C	G	G	G	0.00	0.06	8.78	0.003*	0.25 (0.03–2.20)
5	C	C	C	A	0.04	0.00	7.04	0.008*	0.45* (0.36–0.55)

## Discussion

PE is a hypertensive disorder of pregnancy and characterised by hypertension and proteinuria during gestation. Complications of PE affect 5-8% of pregnancies worldwide and are a leading cause of maternal and infant morbidity and mortality. Multiple factors, including immune activation, endothelial dysfunction, and vascular resistance, play a role in the PE pathophysiology [1] [24]. However, exact underlying relationships between PE and these multiple factors, and pathophysiology of PE remains unknown.

EETs are hyperpolarising vasodilators having anti-inflammatory properties [25]. They contribute to the regulation of uterine blood flow and blood pressure during normal pregnancy and have an important role in the pathogenesis of pregnancy-induced hypertension [19] [20]. It has been shown that plasma EET levels were decreased in PE patients, and it was suggested that they might have crucial effects on systemic and fetoplacental hemodynamics during normal and preeclamptic gestation [18] [26].

Arachidonic acid is oxidised by the CYP monooxygenase to EETs, and sEH rapidly hydrolyses these molecules to the corresponding DHETs, which are far less biologically active than EETs [27]. Thus, alterations in sEH and/or CYP enzyme activities that reduce circulating EET levels may be associated with PE. Many studies showed altered sEH and/or CYP activities in patients with PE and also preeclamptic animal models [18] [28] [29].

It has been found that some genetic variations in the *EPHX2* cause individual differences in sEH activity [30] [31]. Two common SNPs that result in an increase (K55R) or decrease (R287Q) in sEH activity are associated with hypertension [11], CHD [10] [11], and ischemic stroke [11]. Besides, several studies demonstrated that sEH expression levels have an effect on blood pressure by altering the EET levels [32] [33]. Because PE and CHD share many risk factors and pathophysiological features and promoter methylation, that has been shown to affect *EPHX2* expression [34], we hypothesised and investigated for the first time in our previous study that K55R polymorphism and promoter methylation levels in *EPHX2* may be associated with PE. Hence, we concluded that the increase of sEH expression or activity caused by hypomethylation of the *EPHX2* promoter and functional polymorphisms such as K55R respectively were associated with a significantly increased risk of PE [21]. These findings led us to investigate the association between PE and promoter sequence variations that may influence the expression level of the sEH gene. As far as we know, this is the first study to evaluate the relation between *EPHX2* promoter sequence and PE.

Here, we found four SNPs in the promoter region of the *EPHX2* (rs62504268, rs4149232, rs4149235, and rs73227309) that were significantly associated with PE (p < 0.05). Our results demonstrated that pregnant women who carry heterozygous and homozygous polymorphic genotype of the SNPs rs62504268 (GA+AA), rs4149232 (TC+CC), rs4149235 (GC/GA+CC/AA) and rs73227309 (GC+CC) have 2.83, 2.43, 4.38, and 2.29 times increased risk of PE, respectively. Besides, we found a significant linkage disequilibrium between rs73227309 and rs62504268 (D' = 0.908; r^2^ = 0.756), and rs4149232 and rs4149235 (D' = 0.82; r^2^ = 0.645) (Table 4 and Figure 1). Furthermore, in the present study, we successfully established haplotypes for the *EPHX2* from the different combinations of the four SNPs. The haplotype CCGG was associated with increased risk for PE (OR: 3.11; 95% CI: 1.12-8.62) whereas the CCCA haplotype was decreased the risk for the disease (OR: 0.45; 95% CI: (0.36-0.55). Our results suggest that these polymorphisms in the promoter region of the *EPHX2* could be an important risk factor for the development of PE. These remarkable ORs indicate that these variant genotypes in the promoter of *EPHX2* may alter the sEH gene expression. We can say individuals carrying these 4 SNPs and/or haplotype CCGG may have increased sEH and therefore decreased EET levels in the circulation. Increased sEH expression results in an increase in the sEH activity and reduction of the anti-hypertensive, vasodilator, and anti-inflammatory properties of the EET molecules. EETs are important molecules in the pregnancy. Zhou et al. showed that EET synthesis in the kidney is elevated during pregnancy, and downregulation of renal epoxygenase activity by a selective epoxygenase inhibitor causes hypertension in pregnant rats. They reported that EETs may contribute to the control of blood pressure during pregnancy [19]. Catella et al. [20] showed that patients with pregnancy-induced hypertension excreted higher levels of the11,12-DHET and 14,15-DHET than healthy pregnant women, indicating increased EET catabolism in these patients. Consistent with these studies, our previous study also revealed higher 11,12-DHET levels, a representative metabolite of sEH-mediated metabolism of EET, in PE patients compared to the normotensive pregnant women [35]. Our current ndings suggest that these four polymorphisms in the promoter region of the *EPHX2* may lead to a change in the levels of EETs in the circulation of PE patients via affecting the gene expression. Taken all together, one can conclude that reduced EET levels as a result of increased sEH expression or activity may contribute to the increased blood pressure in PE patients. Thus, using of sEH inhibitors may have a therapeutic benefit, especially in PE women who carry polymorphic genotype of the SNPs rs62504268, rs4149232, rs4149235, rs73227309, and also K55R polymorphism.

In conclusion, rs62504268, rs4149232, rs4149235, and rs73227309 polymorphisms in the promoter region of *EPHX2*, and CCGG and CCCA haplotypes were associated with PE. These SNPs may play a role in the pathogenesis of PE by reducing the anti-inflammatory, antihypertensive and vasodilator properties of the EETs via affecting the gene expression. The CCGG haplotype appears to cause an increased risk for PE while the CCCA haplotype may be protective against PE in the Turkish population. However, additional studies are required to support our findings in larger size populations and mechanism-based studies to clarify the effect of these SNPs on the enzyme activity and/or expression levels.

*Acknowledgements*. We are thankful to all thestudy participants for their contribution. This work wassupported by the Scientific Research Project Fund of Sivas Cumhuriyet University under project number T658.

## Conflict of interest statement

All the authors declare that they have no conflict of interest in this work.

## List of abbreviations

CHD, coronary heart disease; CI, confidence intervals; DHETs, dihydroxyeicosatrienoic acids; EETs, epoxyeicosatrienoic acids; LD, linkage disequilibrium; OR, odds ratios; PCR, polymerase chain reaction; PE, Pre eclampsia; sEH, soluble epoxide hydrolase; SNPs, single nucleotide polymorphisms
